# Prognostic implications of PD-L1 expression in patients with soft tissue sarcoma

**DOI:** 10.1186/s12885-016-2451-6

**Published:** 2016-07-08

**Authors:** Chan Kim, Eun Kyung Kim, Hun Jung, Hong Jae Chon, Jung Woo Han, Kyoo-Ho Shin, Hyuk Hu, Kyung Sik Kim, Young Deuk Choi, Sunghoon Kim, Young Han Lee, Jin-Suck Suh, Joong Bae Ahn, Hyun Cheol Chung, Sung Hoon Noh, Sun Young Rha, Soo Hee Kim, Hyo Song Kim

**Affiliations:** Division of Medical Oncology, Department of Internal Medicine, Yonsei University College of Medicine, 134 Shinchondong, Seodaemun-gu, Seoul, 03722 Korea; Department of Pathology, Yonsei University College of Medicine, Seoul, Korea; Oncology, Merck Sharp & Dohme, Seoul, Korea; Department of Pediatric Hemato-Oncology, Yonsei University College of Medicine, Seoul, Korea; Department of Orthopedic Surgery, Yonsei University College of Medicine, Seoul, Korea; Department of Surgery, Yonsei University College of Medicine, Seoul, Korea; Department of Urology, Yonsei University College of Medicine, Seoul, Korea; Department of Obstetrics and Gynecology, Yonsei University College of Medicine, Seoul, Korea; Department of Radiology, Yonsei University College of Medicine, Seoul, Korea; Medical Oncology, CHA Bundang Medical Center, CHA University, Seongnam, Korea; Anatomic Pathology Reference Lab, Seegene Medical Foundation, Seoul, 05548 Korea

**Keywords:** Soft tissue sarcoma, PD-L1, Biomarker, Prognosis

## Abstract

**Background:**

The PD-1/PD-L1 axis plays a paramount role in the immune escape of tumor cells by negative regulation of T-cell functions. The aim of the present study was to characterize the PD-L1 expression pattern and its clinical implication in soft-tissue sarcomas (STS).

**Methods:**

We analyzed PD-L1 expression in 82 STS patients with 5 subtypes: rhabdomyosarcoma, synovial sarcoma, Ewing sarcoma, epithelioid sarcoma, and mesenchymal chondrosarcoma.

**Results:**

The median age at diagnosis was 26 (range: 1–78) and the male to female ratio was 1.6. The majority (80 %) of patients showed locoregional disease rather than metastatic disease at diagnosis. Thirty-five cases (43 %) showed PD-L1 expression and the proportion of PD-L1 expression was significantly different according to histologic subtypes (*P* = 0.004); highest in epithelioid sarcoma (100 %, 7/7), followed by synovial sarcoma (53 %, 10/19), rhabdomyosarcoma (38 %, 12/32), and Ewing sarcoma (33 %, 6/18), while it was not expressed in mesenchymal chondrosarcoma (0 %, 0/6). STS patients with PD-L1 expression had worse overall survival compared with those without PD-L1 expression (5-year survival rate: 48 % *vs*. 68 %, *P* = 0.015). The Cox proportional hazard model adjusted for histologic subtype, initial metastasis, and PD-L1 expression showed that PD-L1 expression was significantly associated with shorter overall survival (*P* = 0.037, HR 2.57, 95 % CI 1.060–6.231).

**Conclusion:**

We have confirmed PD-L1 expression in various STS of young population and demonstrated its independent negative prognostic role, thereby suggesting the PD-1/PD-L1 axis as a potential therapeutic target for the treatment of young STS patients.

**Electronic supplementary material:**

The online version of this article (doi:10.1186/s12885-016-2451-6) contains supplementary material, which is available to authorized users.

## Background

Sarcomas are a rare and highly heterogeneous group of neoplasms originating from the bone and soft tissue, which account for <1 % of all human malignancies [1, 2]. With strikingly variable genetic aberrations, various sarcomas have abnormal fusion proteins arising from translocations. In spite of the multimodality treatments with surgery, radiotherapy, and combination chemotherapy, more than 40 % of cases ultimately experience tumor recurrence, which results in an overall survival (OS) of <12 months [3]. There are still only a few treatment options left when conventional treatment fails; thereby, novel anti-cancer therapeutics are desperately needed to treat these devastating diseases.

It is well known that the prognosis of a malignant tumor is closely related to host immune responses. During the immune response, the priming and activation of T-cells are critical processes in the induction of adaptive immunity, and the ultimate amplitude of the immune response is regulated by a balance between co-stimulatory and inhibitory signals. In this T-cell-mediated process, cytotoxic T-lymphocyte antigen 4 (CTLA-4) provides inhibitory signals in the priming phase of the T-cell response within the lymph node. The programmed death 1 (PD-1) receptor is one of the key inhibitory signals that is induced during the chronic antigen exposure in peripheral tumor microenvironments. The interaction between PD-L1 in tumor cells and PD-1 in T-lymphocytes negatively regulates the effector function of tumor-specific T-lymphocytes and allows tumor cells to evade the host immune system. Recent studies have indicated that high expression of PD-L1 is associated with poor prognosis in non-small cell lung cancer (NSCLC), ovarian cancer, and kidney cancer [4–7]. Furthermore, because anti-PD-1 antibodies were approved in melanoma and lung cancer with robust efficacy and safety profiles, much attention has paid to the PD-L1 expression in various solid malignancies. However, the presence of PD-L1 and its clinical implications in sarcoma have not been widely investigated to date.

In this study, we investigated PD-L1 expression in soft tissue sarcoma and evaluated its clinical relevance according to different subtypes of sarcoma. We thereafter analyzed the prognostic potential of PD-L1 to provide a practical guide as a diagnostic and therapeutic strategy.

## Methods

### Patients and tissue samples

This study was conducted in a retrospective cohort of patients who were pathologically diagnosed with CD99 positive sarcomas such as rhabdomyosarcoma, synovial sarcoma, Ewing sarcoma, epithelioid sarcoma, and mesenchymal chondrosarcoma, between 1994 and 2013 at Yonsei Cancer Center. A total of 82 formalin-fixed, paraffin-embedded tissue blocks were available for examination of PD-L1 expression. All hematoxylin and eosin (H&E) slides were independently reviewed by two experienced pathologists (E.K.K. and S.H.K).

The clinicopathologic variables such as sex, age, maximal tumor size, tumor histology and grade, tumor location, tumor stage, initial presentation of disease, and the status of the resection margin were reviewed retrospectively based on electronic medical records. Tumors were graded according to the French Federation Nationale des Centre de Lutte Contre le Cancer (FNCLCC) criteria [8]. Staging was determined using the 7th edition of the American Joint Committee on Cancer guideline of tumor, node, and metastasis (TNM) classification. The study was approved by the institutional review board of Severance Hospital, Seoul, Korea.

### Tissue microarray (TMA) construction

Simple, inexpensive, and precise paraffin TMAs were constructed with a conventional microcompound table and a drill grinder. The original H&E slides were reviewed by two pathologists (E.K.K and S.H.K). One or two different tumor areas per case were selected for tissue microarray construction. Core tissue biopsies (3 mm in diameter) were taken from the individual paraffin blocks (donor blocks) and arranged in recipient paraffin blocks (tissue array blocks) using a trephine apparatus. All TMA blocks were confirmed by H&E staining.

### Immunohistochemical staining and assessment

Immunohistochemistry (IHC) staining of PD-L1 was performed on the TMA blocks using a mouse monoclonal antibody for PD-L1 (clone 130021, R&D Systems, Minneapolis, MN). Four-micrometer-thick sections were prepared and stained using a Ventana automatic immunostainer (Ventana, Benchmark, Tucson, AZ) [9]. After deparaffinization, heat-induced antigen retrieval was performed using pH 6.0 citrate buffer (CC1 protocol, Ventana, Tucson, AZ), and reactivity was detected using the Cell Mark detection kit (catalog no. 315-M-94). The sections were incubated with the primary antibody for 32 min at room temperature (dilution 1:100). The slides were counterstained with hematoxylin. Positivity for PD-L1 was evaluated and determined independently by two experienced pathologists (E.K.K. and S.H.K). In the case of discrepancy, a consensus was made through an in-depth discussion on multi-head microscopic observations. The intensity and percentage of PD-L1 positive tumor cells were counted manually in at least 100 viable tumor cells from 4 representative fields in each case. Because there was no consensus on the scoring system for PD-L1 expression, especially in sarcoma, we modified the previous protocol [10] and describe PD-L1 expression as much detail as possible by a semi-quantitative manner using the intensity multiplied by the proportion. The staining intensity was graded as negative (0), weak to moderate (1), or strong (2), and the proportion was categorized by the percentage of positive cells as 0, no positive tumor cells; 1+, less than 10 %; 2+, 10–50 %; and 3+, >50 %. Based on the multiplication score, a score of 0 or 1 was considered as negative PD-L1 expression, whereas scores greater than 2 were considered positive PD-L1 expression.

### Statistical methods

The Statistical Package for the Social Sciences (SPSS) version 18.0 (IBM, Chicago, IL) was used for statistical analyses. The correlations between PD-L1 expression and clinicopathologic variables were analyzed using the independent sample *t*-test for the continuous variables and the chi-square test for the discrete variables. For survival analysis, recurrence-free survival (RFS) was defined as the time interval between surgery and tumor recurrence or last follow-up. OS was defined as the time interval between the diagnosis of metastatic/recurrent disease and death or last follow-up. Survival analysis was performed using the Kaplan-Meier method with the log-rank test. Multivariate analyses for OS were performed with Cox’s regression. The accepted level of statistical significance was *P* < 0.05.

## Results

### Patient characteristics

The baseline clinicopathologic characteristics of the patients are summarized in Table 1. The male-to-female ratio was 1.6:1, and the median age at the time of diagnosis was 26 years (range 1–78). The median tumor size was 5 cm, and two-thirds of cases showed low- or intermediate-grade tumors. Approximately half of the cases were rhabdomyosarcoma (*n* = 32, 39 %), followed by synovial sarcoma (*n* = 19, 23 %), Ewing sarcoma (*n* = 19, 22 %), epithelioid sarcoma (*n* = 7, 9 %), and mesenchymal chondrosarcoma (*n* = 6, 7 %). One third of the tumors were located in the extremities (*n* = 27, 33 %). Most patients had no distant metastasis at the time of diagnosis (82 %) and underwent surgical resection (93 %). Adjuvant chemotherapy was administered to 47 patients (57 %), and 37 of these patients (79 %) also received adjuvant radiotherapy.

**Table 1:** Baseline characteristics

Variables			PD-L1 expression		*P* value
		N (%)	-	+	
Sex	Male	50 (61 %)	31 (66 %)	19 (54 %)	0.284
	Female	32 (39 %)	16 (34 %)	16 (46 %)	
Age (year, median, range)		26 (1–78)	26.3 ± 19.7	30.6 ± 20.0	0.773
Tumor size (cm, median, range)		5.0 (1.5–12.0)	5.0 ± 1.8	5.3 ± 1.7	0.711
Histologic type	Rhabdomyosarcoma	32 (39 %)	20 (43 %)	12 (34 %)	0.004
	Synovial sarcoma	19 (23 %)	9 (19 %)	10 (29 %)	
	Ewing sarcoma	18 (22 %)	12 (26 %)	6 (17 %)	
	Epithelioid sarcoma	7 (9 %)	0 (0 %)	7 (20 %)	
	Mesenchymal chondronsarcoma	6 (7 %)	6 (13 %)	0 (0 %)	
Tumor location	Trunk	13 (16 %)	9 (19 %)	4 (11 %)	0.073
	Abdomen/pelvis	20 (24 %)	14 (30 %)	6 (17 %)	
	Head/Neck	22 (27 %)	14 (30 %)	8 (23 %)	
	Extremities	27 (33 %)	10 (21 %)	17 (49 %)	
FNCLCC Grade	1	17 (21 %)	11 (23 %)	6 (17 %)	0.242
	2	34 (41 %)	19 (40 %)	15 (43 %)	
	3	31 (38 %)	17 (36 %)	14 (40 %)	
Initial distant metastasis	No	67 (82 %)	38 (81 %)	29 (83 %)	0.816
	Yes	15 (18 %)	9 (19 %)	6 (17 %)	
Surgery	No	6 (7 %)	2 (4 %)	4 (11 %)	0.217
	Yes	76 (93 %)	45 (96 %)	31 (89 %)	
Resection margin	Negative	59 (72 %)	29 (62 %)	30 (86 %)	0.017
	Positive	23 (28 %)	18 (38 %)	5 (14 %)	
Chemotherapy	No	35 (43 %)	21 (45 %)	14 (40 %)	0.672
	Yes	47 (57 %)	26 (55 %)	21 (60 %)	
Radiotherapy	No	45 (55 %)	26 (55 %)	19 (54 %)	0.926
	Yes	37 (45 %)	21 (45 %)	16 (46 %)	

### PD-L1 expression status and clinicopathologic features

**Fig. 1:**
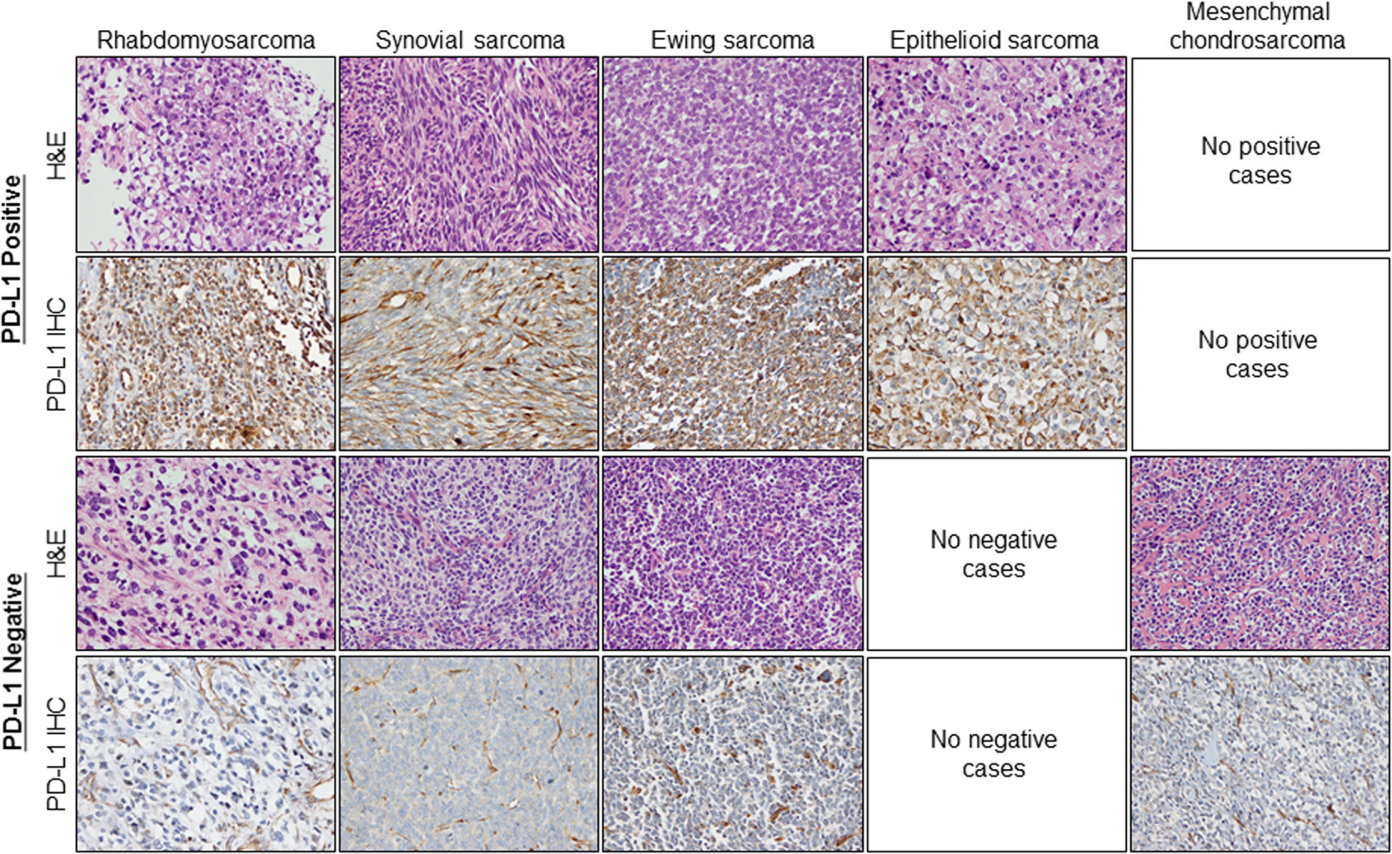
Immunohistochemical staining of PD-L1 expression. Representative images of PD-L1-positive and -negative sarcoma samples

Among the 82 sarcoma patients, PD-L1 was expressed in 35 cases (43 %). Representative images of PD-L1-positive and -negative staining for each histologic type are shown in Fig. 1. In the positive cases, PD-L1 was positive in the cytoplasmic membrane of the tumor cells and intratumoral endothelial cells. PD-L1 expression was significantly different according to the histologic subtype of sarcoma (*P* = 0.004). The proportion of PD-L1-expressing tumors was highest in epithelioid sarcoma (100 %, 7/7), followed by synovial sarcoma (53 %, 10/19), rhabdomyosarcoma (38 %, 12/32), and Ewing sarcoma (33 %, 6/18), whereas it was not expressed in mesenchymal chondrosarcoma (0 %, 0/6). Patients with PD-L1 expression had a more negative resection margin than the PD-L1-negative group (86 % vs*.* 62 %, *P* = 0.017). There was no significant difference in PD-L1 expression regarding age, sex, tumor size, location, histologic grade, surgical resection, and adjuvant treatment.

### Survival outcome according to PD-L1 expression

**Fig. 2:**
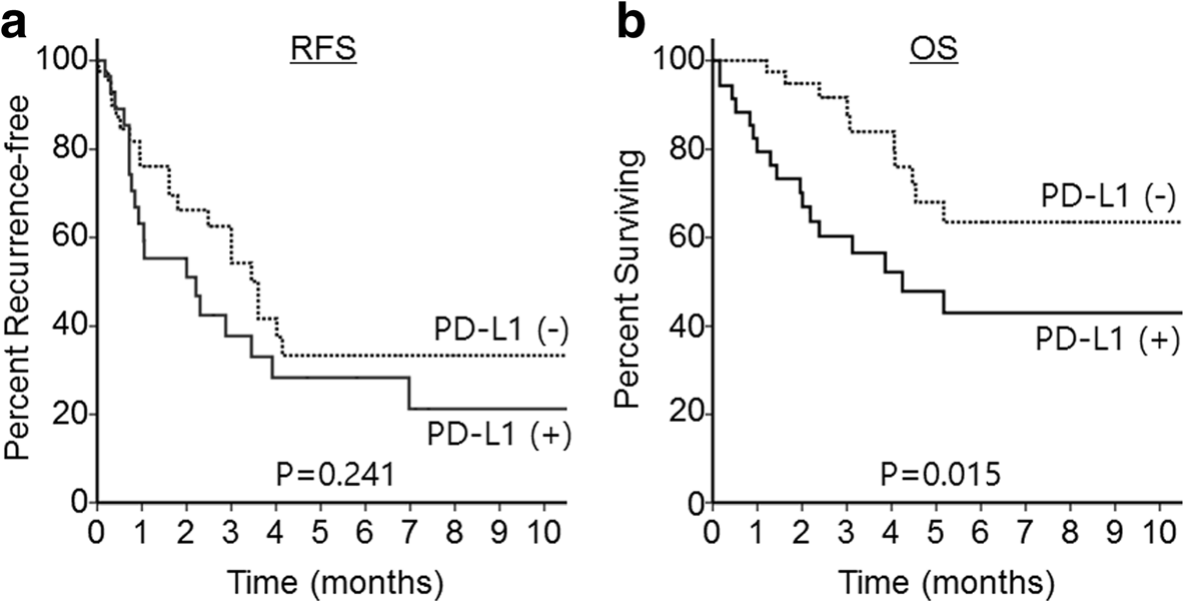
Survival analyses according to PD-L1 expression. **a** Kaplan-Meier survival curves for recurrence-free survival (RFS). **b** Kaplan-Meier survival curves for overall survival (OS)

After a median follow-up duration of 33.8 months, 27 patients (33 %) had died at the time of survival analysis. The 5-year OS for all patients was 58.8 % and the 5-year OS rates of the subgroups were as follows: 64 % for rhabdomyosarcoma, 77 % for synovial sarcoma, 39 % for Ewing sarcoma, 18 % for epithelioid sarcoma, and 100 % for chondrosarcoma. There was a trend toward a worse RFS for the positive PD-L1 expression group compared to the negative group (Fig. 2a). Expression of PD-L1 in the tumor tissue significantly predicted shortened OS (5-year OS rate, 48 % vs. 68 %; hazard ratio [HR] = 2.545; 95 % confidence interval [CI] = 1.16–5.56; *P* = 0.015, Fig. 2b). Because the study cohort includes patients with initial distant metastasis and this could influence the survival outcome, we also analyzed the RFS and OS after excluding patients with metastatic disease. The trend for RFS and OS were almost comparable whether we exclude or not the metastatic patients (Additional file 1: Figure S1). The clinicopathologic variables significantly correlated with OS by univariate analysis were histologic subtype, initial metastasis, and PD-L1 expression (Table 2). In the Cox proportional hazard model adjusted for histologic subtype, initial metastasis, and PD-L1 expression, PD-L1 expression was significantly associated with shorter OS (*P* = 0.037, HR = 2.57, 95 % CI = 1.06–6.23).

**Table 2:** Univariate and multivariate analysis for overall survival

Variables	Univariate		Multivariate	
	5-years OS	*P*-value	HR (95 % CI)	*P*-value
Sex (Male vs. Female)	63 % vs. 52 %	0.553		
Age (≥20 vs. <20)	60 % vs. 57 %	0.871		
Tumor size (≥5 cm vs. <5 cm)	53 % vs. 65 %	0.136		
Histologic type (Epi. Sarcoma vs. Others)	18 % vs. 63 %	0.004	1.459 (0.414–5.137)	0.556
Tumor location (Axial vs. Extremity)	57 % vs. 63 %	0.423		
Initial metastasis (Yes vs. No)	33 % vs. 65 %	0.034	2.335 (0.858–6.351)	0.097
Surgery (Yes vs. No)	62 % vs. 40 %	0.069		
Margin status (Positive vs. Negative)	51 % vs. 62 %	0.819		
Adjuvant therapy (Yes vs. No)	58 % vs. 65 %	0.921		
PD-L1 expression (Yes vs. No)	48 % vs. 68 %	0.015	2.490 (1.032–6.007)	0.042

*HR* hazard ratio, *CI* confidence interval

## Discussion

In this study, we evaluated the clinical relevance of PD-L1 expression in various sarcoma subtypes. PD-L1 was differently expressed according to the histologic subtypes of sarcoma and it was found to be an independent prognostic factor for OS.

William Coley, a bone surgeon at New York Memorial Hospital, depicted the spontaneous tumor regression of sarcoma patients after severe bacterial infection more than 100 years ago [10, 11]. Subsequently, there were several reports related to the cure of metastatic sarcoma with aggressive surgical resection, which suggested a potential therapeutic role of immune surveillance [12]. Since then, the interest in immunotherapy for sarcoma treatment has risen and fallen. Hypothetically, because several types of sarcoma have a common and specific chromosomal translocation, the resulting fusion proteins may be potential tumor neoantigens that could be appropriate targets for immunotherapy [13].

Based on this theoretical background, numerous clinical trials were attempted in sarcoma patients with various immunomodulatory agents such as macrophage-colony stimulating factor (GM-CSF), peptide vaccines, and anti-CTLA-4 antibody [14–16]. Inhaled GM-CSF was introduced for 43 patients with first isolated pulmonary recurrence of osteosarcoma [14]. Although this treatment seemed feasible with low toxicity, no immunomodulatory effect or improved outcome was observed. Another study with the anti-CTLA-4 antibody ipilimumab for synovial sarcoma was halted due to poor accrual and no clinical response [16]. Besides these disappointing results in previous clinical trials, there have been few studies exploring potential therapeutic targets for immunotherapy in sarcoma conducted to date.

Recently, with the impressive and outstanding success of pembrolizumab and nivolumab in melanoma, NSCLC, and other malignancies [17, 18], immune checkpoint inhibitors have come into the limelight for the treatment of various solid tumors. Although numerous trials of PD-1/PD-L1 inhibitors are ongoing for various solid tumors, there has been minimal research to investigate the clinical significance of the PD-1/PD-L1 axis in sarcoma. In the present study, we revealed that 42.7 % of the sarcoma patients had positive expression of PD-L1, which varied according to histologic subtypes. Epithelioid and synovial sarcoma had higher positive expression rates (100 and 53 %), whereas mesenchymal chondrosarcoma cases revealed no PD-L1 expression. This finding suggests that PD-L1 expression is also heterogeneous according to different histologic subtypes of sarcoma, and that PD-L1 blockade could be a novel and promising therapeutic strategy in this orphan tumor.

The PD-1/PD-L1 expression level has been reported to be related to poor survival in other solid tumors. In a previous report of renal cell carcinoma, PD-1 expression in tumor-infiltrating lymphocytes was observed in half of the cases and was associated with poor survival [7]. Moreover, PD-L1 expression was reported in half of the gastric cancer and lung cancer cases and was an independent negative prognostic factor for OS [19, 20]. In the present study, in addition to determining the frequency of PD-L1 expression according to histologic subtypes, we were able to demonstrate the prognostic role of the intratumoral PD-L1 expression in sarcoma. PD-L1 was significantly associated with shorter 5-year OS regardless of sex, age, tumor size, histology, location, surgical outcome, and adjuvant treatment, implying that PD-L1 is an independent negative prognostic factor in sarcoma.

Moreover, besides the prognostic value of PD-L1 expression in human cancers, it is becoming increasingly recognized as an important biomarker for predicting the treatment efficacy of PD-1/PD-L1 blockade. In the KEYNOTE-012 phase IB trial (Clinical Trials.gov Identifier; NCT01848834) [21], patients with advanced gastric cancer were screened for PD-L1 expression (positivity was defined as PD-L1 expression in ≥ 1 % of cells in tumor nests or according to stromal staining using IHC with the 22C3 PD-L1 antibody), and 41 % were PD-L1-positive cases. After treatment with an anti-PD-1 antibody, pembrolizumab, a promising objective response rate (22 %), 6-month progression free survival (26 %), and 6-month OS (66 %) were observed. Furthermore, Herbst et al. [22] reported the association of PD-L1 expression in tumor-infiltrating immune cells with the response to an anti-PD-L1 monoclonal antibody, MPDL3280A, in which 83 % of IHC 3+ NSCLC cases showed a response, whereas the response rate was less than 20 % in IHC 0–1+ cases. In contrast to the above-stated findings, the survival outcome for another anti-PD-1 antibody, nivolumab, was not significantly different according to PD-L1 subgroup in melanoma patients [23]. Taken together, the predictive value of PD-L1 expression under treatment with PD-1/PD-L1 immune checkpoint inhibitors has not yet been fully established, and therefore further validation is strongly warranted through further studies.

Unfortunately, the IHC criteria for PD-L1 expression have not yet been standardized. Indeed, different definitions of positive PD-L1 expression have been used in the clinical trials conducted to date. In the CheckMate 017 study with NSCLC patients, OS and response rate were significantly better with nivolumab than with docetaxel, regardless of the PD-L1 expression level [17]. PD-L1 expression (of tumor cells only) was neither prognostic nor predictive of nivolumab efficacy in the study. A prospective study with pembrolizumab, KENOTE-010, confirmed the clinical usefulness of the tumor proportion score (PD-L1 expression in at least 1 % of tumor cells) [24]. Considering that patients with a higher tumor proportion score (≥50 %) had a significantly increased benefit compared to those with a lower score (≥1 %), further studies are required to determine the appropriate cutoff value of the proportion score. Furthermore, because previous studies reported marked intra-patient discordance and longitudinal heterogeneity of PD-L1 assays, rigorous validation with clinical trials are still needed [25]. Comprehensive incorporation with tumor-infiltrating cells [26], inflammatory gene signatures [27], or the immune microenvironment [28] will be helpful to improve patient identification. Currently, a blueprint project to comprehensively compare various PD-L1 assay are ongoing by FDA and ASCO (Sholl et al. Arch Pathol Lab Med 2016), we can wait these results to answer this important question.

The main limitations of our study include its patient selection, small sample size, and imbalance of histologic type. Because we initially intended to analyze the PD-L1 expression in CD99 positive tumors, this study mainly enrolled pediatric STS, which could result in relatively younger median age of patients and longer survival outcome. Moreover, due to the small sample size and imbalance of histologic type between PD-L1 positive and negative groups, especially epithelioid sarcoma and mesenchymal chondrosarcoma, there could be a possibility of type I error. Therefore, our findings should be validated in an independent STS cohort and according to the response to PD-1/PD-L1 inhibitors in future clinical trials.

## Conclusion

In conclusion, we have revealed PD-L1 expression in various STS of young population and demonstrated its independent negative prognostic role, thereby suggesting the PD-1/PD-L1 axis as a potential therapeutic target for the treatment of young STS patients.

## Abbreviations

CTLA-4, cytotoxic T-lymphocyte antigen 4; IHC, immunohistochemistry; OS, overall survival; PD1, programmed death 1; PD-L1, programmed death ligand 1; RFS, recurrence-free survival; TMA, tissue microarray

## Additional file

Additional file 1: Figure S1. Survival analyses according to PD-L1 expression in patients with localized disease. (A) Kaplan-Meier survival curves for recurrence-free survival (RFS). (B) Kaplan-Meier survival curves for overall survival (OS). (TIF 84 kb)
