# Supplementary figures and images for: Prognostic implications of PD-L1 expression in patients with soft tissue sarcoma
Source: BMC Cancer. 2016 Jul 8;16:434. doi: 10.1186/s12885-016-2451-6 (PMC4938996; doi:10.1186/s12885-016-2451-6)

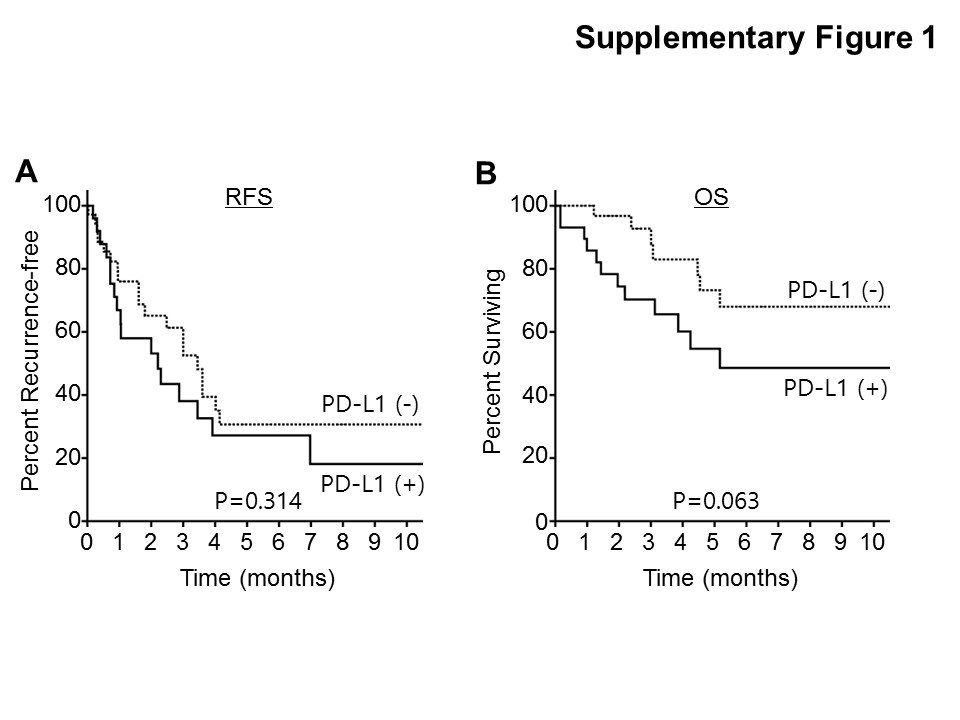

Supplement: Additional file 1: Figure S1. — Survival analyses according to PD-L1 expression in patients with localized disease. (A) Kaplan-Meier survival curves for recurrence-free survival (RFS). (B) Kaplan-Meier survival curves for overall survival (OS). (TIF 84 kb) [file 12885_2016_2451_MOESM1_ESM.tif]
